# A Lightweight Deep Learning Network on a System-on-Chip for Wearable Ultrasound Bladder Volume Measurement Systems: Preliminary Study

**DOI:** 10.3390/bioengineering10050525

**Published:** 2023-04-26

**Authors:** Hyunwoo Cho, Ilseob Song, Jihun Jang, Yangmo Yoo

**Affiliations:** 1 Department of Electronic Engineering, Sogang University, Seoul 04107, Republic of Korea; hyunwoocho@sogang.ac.kr; 2 Medical Solutions Institute, Sogang University, Seoul 04107, Republic of Korea; ilseob@sogang.ac.kr; 3 Edgecare Inc., TE1103, 35 Baekbeom-ro, Mapo-gu, Seoul 04107, Republic of Korea; 4 Department of Biomedical Engineering, Sogang University, Seoul 04107, Republic of Korea

**Keywords:** deep learning, semantic segmentation, automatic volume measurement, ultrasound bladder scanner, edge computing, urinary disease

## Abstract

Bladder volume assessments are crucial for managing urinary disorders. Ultrasound imaging (US) is a preferred noninvasive, cost-effective imaging modality for bladder observation and volume measurements. However, the high operator dependency of US is a major challenge due to the difficulty in evaluating ultrasound images without professional expertise. To address this issue, image-based automatic bladder volume estimation methods have been introduced, but most conventional methods require high-complexity computing resources that are not available in point-of-care (POC) settings. Therefore, in this study, a deep learning-based bladder volume measurement system was developed for POC settings using a lightweight convolutional neural network (CNN)-based segmentation model, which was optimized on a low-resource system-on-chip (SoC) to detect and segment the bladder region in ultrasound images in real time. The proposed model achieved high accuracy and robustness and can be executed on the low-resource SoC at 7.93 frames per second, which is 13.44 times faster than the frame rate of a conventional network with negligible accuracy drawbacks (0.004 of the Dice coefficient). The feasibility of the developed lightweight deep learning network was demonstrated using tissue-mimicking phantoms.

## 1. Introduction

Bladder volume measurements are commonly used in managing urinary diseases, such as urinary incontinence and benign prostate enlargement. Urinary catheterization is often used for measuring bladder volume in many cases, e.g., postoperative urinary retention [1], but it yields a high risk of urinary tract infection. To minimize unnecessary urinary catheterization, several studies have been conducted to analyze the impact and proper cycles of urinary catheterization [2,3]. Measuring post-void residual urine (PVR) is regarded as an effective way to reduce unnecessary catheterization. Additionally, PVR is a useful predictor of various diseases, such as prostatism and urinary tract infection [4,5]. To maximize the advantages of PVR measurements, a fast and accurate PVR measurement method is needed.

Ultrasound imaging (US) is a noninvasive, cost-effective, and real-time imaging modality that has been shown to be one of the most accurate and effective methods for measuring PVR [6,7,8,9]. Several studies have also demonstrated that US can potentially be used in point-of-care (POC) settings [10,11,12]. Recently, the development of portable US imaging devices for measuring bladder volume has been proposed [13,14]. Despite its usefulness, US has several limitations for measuring PVR in POC settings. One of the most challenging problems is its high operator dependency, which makes interpreting ultrasound images without professional experience and expertise difficult. Additionally, in POC settings, limited resources such as computing power and less experienced clinicians (e.g., nurses and care providers) can be problematic. As a result, the ultrasound image quality may be degraded, leading to misinterpretation or difficulties in PVR analysis.

To decrease operator dependency, an automatic bladder volume measurement method is needed. Traditionally, mechanical ultrasound scanning systems, such as a wobbling probe, have been used for PVR measurements. However, these methods require a prescan process to allocate the probe to the proper location before an actual volume measurement. Additionally, with mechanical scanning systems, ultrasound images or bladder volume measurements cannot be carried out in real time, resulting in inefficient repetitions of measurements. Moreover, patient motion during long scanning times may cause errors in PVR measurements. To address these issues, the need for a real-time image-based bladder measurement system has emerged.

To measure bladder volumes in real time, several studies have introduced image-based bladder volume measurements using various image analysis techniques, such as segmentation. Recent advances in deep learning and computer vision techniques have shown promising results for various tasks, including segmentation of regions of interest (e.g., organs and masses) in ultrasound images. In addition, deep learning techniques have been applied for analyzing urine in ultrasound images [15,16,17]. While these studies have shown that deep learning models can accurately segment the bladder and measure PVR volume, these tasks were primarily conducted on highly complex computing resources such as graphic processing units (GPUs). Additionally, in previous studies, ultrasound images were acquired by commercial cart-based ultrasound systems. In contrast, in POC settings, ultrasound images are collected by portable ultrasound systems so the imaging quality may be degraded due to the compactness and low computational power of these systems. This may reduce the accuracy of PVR measurements with deep learning models.

In this study, to address this issue, a lightweight deep-learning model for a portable bladder volume measurement system is proposed. Our proposed system was designed to detect PVR and segment the bladder region in ultrasound images with much fewer parameters; subsequently, an algorithm was employed to automatically measure the bladder volume using the segmentation results. Additionally, considering system integration, to improve its execution time in portable settings, the developed deep learning model was optimized with a fixed-point quantization technique. As a result, the optimized model could measure the bladder volume accurately with fewer than 1 million parameters on a low-resource SoC at high frame rates. The feasibility of our proposed automatic bladder volume measurement system was demonstrated by using various tissue-mimicking phantoms.

## 2. Materials and Methods

### 2.1. Data Acquisition from the Portable Ultrasound System

**Figure 1:**
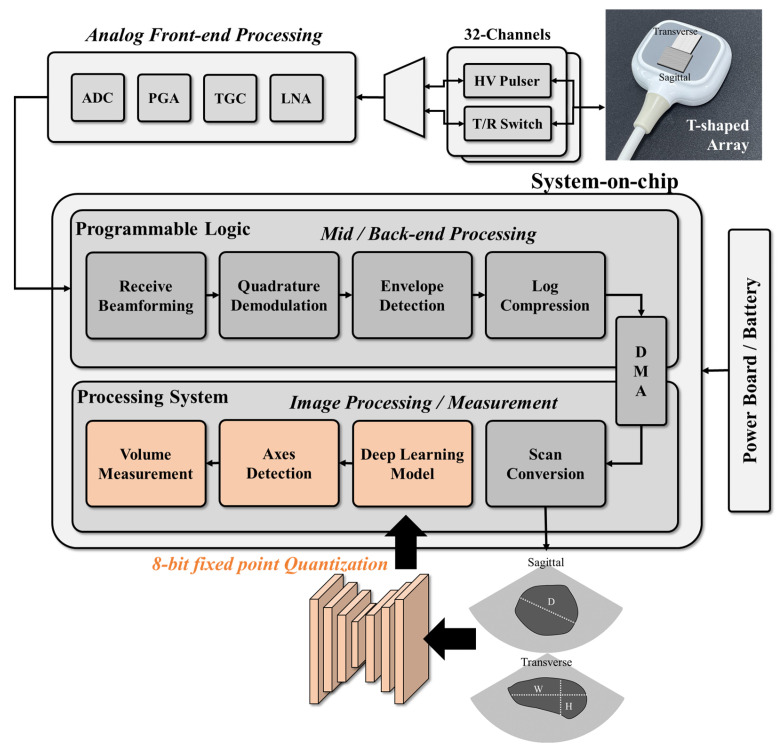
Block diagram showing the processing chains of the integrated system for ultrasound image acquisition and analysis. The gray boxes indicate the original processing blocks of the device, and the orange boxes denote the integrated processing blocks of this study. The deep learning model, trained on biplane images, is implemented on the SoC for bladder segmentation and post-void residual (PVR) detection, enabling automatic bladder volume measurement. To optimize performance, the deep learning model is quantized.

In this study, a system-on-chip (SoC)-based portable ultrasound system (EdgeFlow UH10w, Edgecare Inc., Seoul, Republic of Korea) was used to acquire ultrasound bladder images for training and validating the designed deep learning model. As shown in Figure 1, the commercial ultrasound system includes a SoC, a front-end processing module, and a power module and uses two 32-channel high-voltage (HV) pulsers and a transmit/receive (T/R) switch to control the cross-array probe. Front-end processing involves low noise amplification, time gain compensation, programmable gain amplification, and analog-to-digital conversion. Back-end processing is performed using the programmable logics (PL) on the SoC, with data transfer to the processing system (PS) via a direct memory access (DMA) engine. The signal is then reconstructed into an image using digital scan conversion. To acquire sagittal and transverse images simultaneously, a T-shaped array consisting of two phased array probes was used with the portable ultrasound system. The received radio-frequency signals were processed in PL in the SoC (Zynq Ultrascale+, Xilinx Inc., San Jose, CA, USA) by performing receive beamforming, quadrature demodulation, envelope detection, and log compression. The processed signal was then reconstructed into an image with a height of 330 pixels and a width of 570 pixels by the PS.

The proposed bladder measurement method, based on deep learning, was developed to be integrated into a portable ultrasound system using a system-on-chip (SoC). As depicted in Figure 1, the deep learning network was designed to perform segmentation and classification on the ultrasound images after digital scan conversion (DSC), identifying regions of interest (ROIs) and detecting the bladder. Once the bladder is detected on the image, the bladder volume is estimated by using the length of the axes.

**Figure 2:**
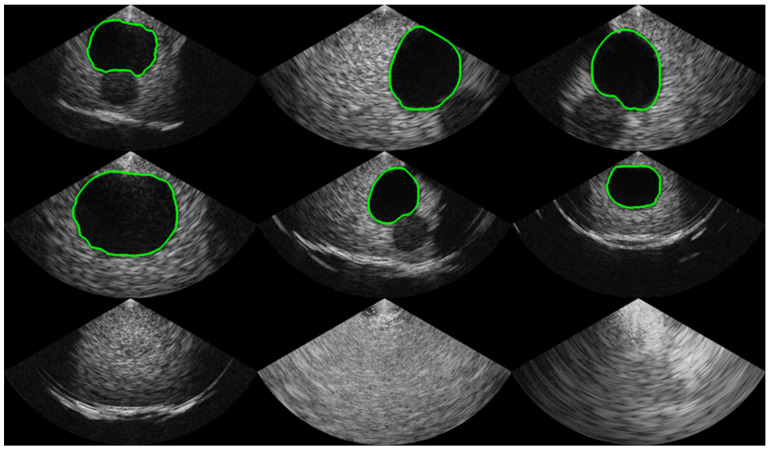
Examples of the acquired dataset. The first and second rows show images with a bladder, and the green line indicates the boundary of the mask label from human labelers. The third row represents images without a bladder, indicating that the bladder was not observed.

To collect a dataset with high variability, various gain and depth settings were used. The ultrasound bladder images were obtained from two tissue-mimicking phantoms: an intravesical urine volume measurement phantom (US-16, Kyoto Kagaku, Kyoto, Japan) with urine volumes of 50 mL, 150 mL, and 300 mL, and a multimodality pelvic phantom (Model 048A, CIRS, Norfolk, VA, USA). A total of 1306 images with a bladder and 2095 images without a bladder were collected. The bladder images were randomly divided into 1044 images for training and 262 images for validation, with each image labeled with a corresponding mask for the segmentation task. The images without a bladder were divided into 1675 images for training and 420 images for validation for the classification task. To capture ROIs of various sizes, the dataset was collected by randomly selecting locations with a free hand on a static phantom. To validate the size and distribution of the dataset, the accuracies on the training phase and validation phase are compared. Examples of the dataset are shown in Figure 2.

### 2.2. Multitask Deep Learning-Based Bladder Volume Measurement

**Figure 3:**
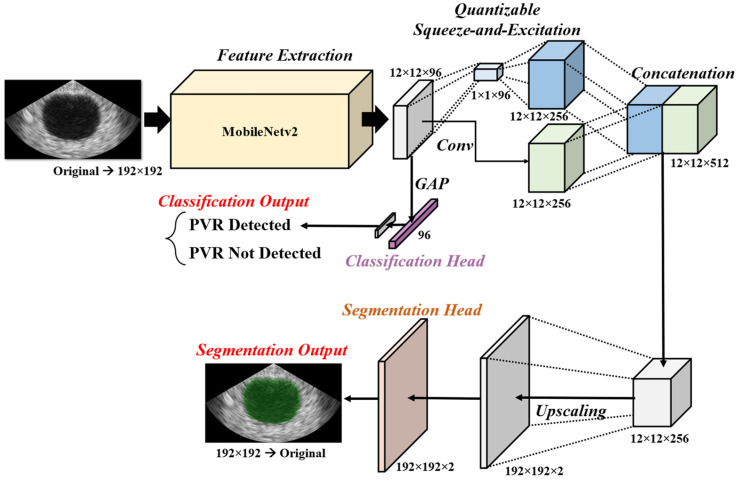
Overall architecture of the deep learning network designed for ultrasound image analysis. The original image is resized to a height and width of 192 pixels before feature extraction using MobileNetv2. The extracted feature is processed through two paths: segmentation and classification. The segmentation path uses squeeze-and-excitation and convolution to expand the features to a dimension of 12 × 12 × 256, which are then concatenated to 12 × 12 × 512 dimensions. A depthwise separable convolution is then applied to merge the gathered feature to a dimension of 12 × 12 × 256 before upscaling it to the 192 × 192 dimension using bilinear interpolation. The channel of the upscaled feature is then reduced to match the number of classes before finalizing segmentation with SoftMax. The classification path uses global average pooling to gather features that are reduced into logits according to each class. The classification is then finalized using SoftMax function.

The aim of this study was to design a convolutional neural network (CNN)-based deep learning model that is simple yet efficient for bladder volume measurement systems. The model was designed to perform multiple tasks, including classification and segmentation, as shown in Figure 3. This multitask approach can improve the model’s efficiency in SoC environments and prevent unexpected measurement results from images without bladder regions. The classification path of the model detects a bladder on the ultrasound image by classifying the image into two classes, indicating the existence of the bladder in the image. The segmentation path of the model aims to find the pixelwise accurate ROI of the bladder in the image. The architecture of the model, including parameters such as the kernel size, was optimized on the collected dataset. Starting from the large-size model, parameters were gradually reduced by comparing the accuracy to the validation dataset.

To reduce the complexity of calculation and memory consumption, the input image was resized to a height and width of 192 pixels. For the feature extraction stage, MobileNetv2 [18], known for its lightweight network architecture and efficiency with regard to portable devices, was used to generate features with dimensions of 12 × 12 × 96. For the classification path, the extracted features were optimized by global average pooling, a dense layer, and classification head layers. For the segmentation path, the features were further processed by quantizable squeeze-and-excitation (QSE) blocks, depthwise separable convolution (DWC) blocks [19], upscaling layers, and a segmentation head. The squeeze-and-excitation (SE) [20] block has been widely used to embed channel weights into features. However, the SE block is not suitable for quantized networks due to elementwise multiplications. Therefore, in this study, a QSE block was designed with a channel weight operation using concatenation and convolution instead of elementwise multiplication. The QSE block was used to capture the larger context of the image. In the SE block, the feature was reduced to a small size vector (1 × 1 × 96) by averaging, and then the expanded feature was weighted by the reduced vector. To merge detailed information with the features from the QSE blocks, 3 × 3 convolution layers were placed parallel to the SE blocks. The features from the QSE blocks and convolution layers were merged by DWC blocks. The merged features were reduced into a smaller channel by convolution layers and then upscaled into the resized input size (192 × 192) with two channels (i.e., the number of pixel classes). Then, the segmentation head classified each pixel into two classes (i.e., background and bladder). Finally, the segmentation result was resized to the original size of the image.

**Figure 4:**
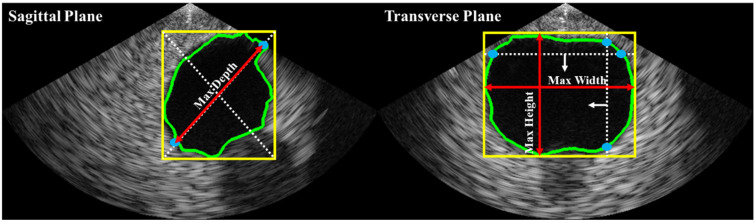
Automated axes detection for estimating bladder height, width, and depth from biplane ultrasound images. The bladder ROI dimensions were obtained by calculating the minimum enclosing bounding box (yellow line). In the sagittal plane, the greater distance between two intersections (blue dots) of the bounding box’s diagonal line (white dashed lines) with the bladder ROI contour (green line) was used to estimate depth. The red arrow lines represent the estimated depth. In the transverse plane, the maximum distances of the vertical and horizontal intersections were used to estimate height and width, respectively.

Bladder volume is typically estimated based on shape coefficients and measurements of height, width, and depth on two different planes (i.e., sagittal and transverse). In this study, as illustrated in Figure 4, depth was estimated on the sagittal plane, while height and width were estimated on the transverse plane. The bladder volume was then calculated using Equation (1), where c is a constant determined by the shape of the bladder region (e.g., 0.52 for a spherical shape, 0.7 for an unknown shape [21]).

Volume ≈ c × Depth × Height × Width(1)

### 2.3. Network Compression and System Implementation

**Figure 5:**
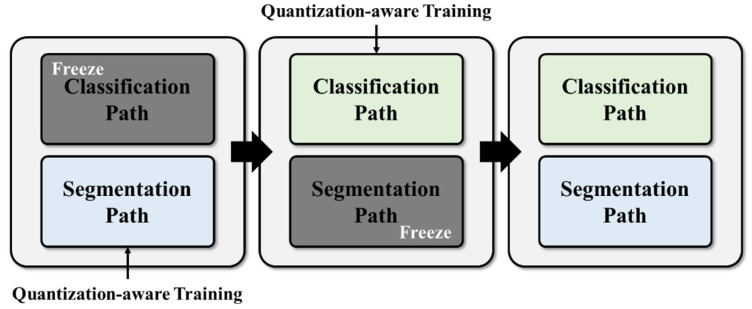
Training process for the designed network. The segmentation path is first trained in a quantization-aware training (QAT) setting with the classification path frozen. Once the segmentation path training is complete, the classification path is trained in the same QAT setting with the segmentation path frozen. Finally, the trained weights from both segmentation and classification paths are combined into a single model.

To train the multitask architecture model without any degradation in accuracy, the classification path and the segmentation path were trained separately. Figure 5 illustrates the three distinct training stages of the proposed network. In the first stage, the segmentation path was trained with the initial weight of the model while the classification path was kept frozen. Next, in the second stage, the segmentation path was frozen, and the classification path was trained. Once the training of the classification path was completed, the weights from the first and second stages were merged into a single model.

Additionally, during the training process, the model was also subjected to quantization-aware training (QAT) [22] to enhance execution speed while minimizing any drop in accuracy. The combo loss function [23], which combines the Dice loss and cross-entropy loss functions, was used to train the segmentation path. Meanwhile, the classification path was trained using the cross-entropy loss function. To avoid overfitting, data augmentation techniques, such as random intensity shift and random left–right flip, were applied. Furthermore, early stopping criteria were implemented, with a patience of 20 epochs. The Adam optimizer [24] was employed to train the network. To avoid local minimum and overfitting problem, the learning rate scheduling and early stopping criterion were used.

The deep learning model is trained using the TensorFlow (Google Inc., Mountain View, CA, USA) framework. After training, the model is compressed to enhance execution time on low-resource SoC settings. Model weights are quantized into 8-bit fixed-point using TensorFlow Lite (Google Inc., Mountain View, CA, USA). Inference is performed using the C++ programming language. To handle the entire system, the Linux operating system is utilized on the SoC with the Vitis (Xilinx Inc., San Jose, CA, USA) framework.

## 3. Results

### 3.1. Evaluation of the Trained Deep Learning Model

For the evaluation of the model’s performance, the segmentation was assessed using the Dice coefficient metric, while accuracy was used to evaluate the classification performance. To compare the segmentation results with conventional models, U-Net [25], Attention U-Net [26], and BiSeNetv2 [27] were implemented at the original image resolution of 570 × 330. U-Net and Attention U-Net were implemented with 64, 128, 256, and 512 channels. The same optimizer and loss function were used for both the proposed and conventional methods. The conventional methods were trained and implemented using a 32-bit floating point, while the proposed method was trained and implemented using an 8-bit fixed point, as previously mentioned. The models were compared on the same validation data. The results are presented in Table 1, where F and Q represent 32-bit floating point and 8-bit fixed point implementation, respectively. The throughput in Table 1 was measured in the integrated SoC setting.

**Figure 6:**
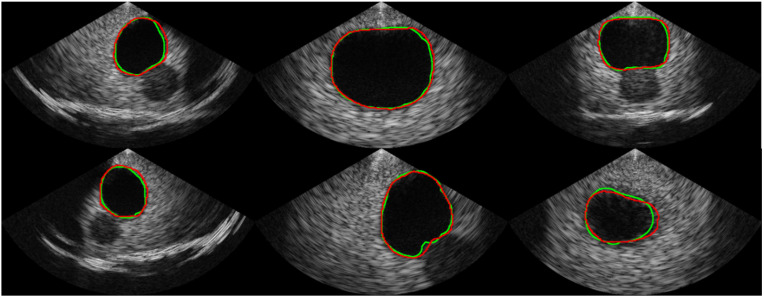
Examples of the segmentation result. The ground truth is represented by the green line, while the prediction from our proposed network is represented by the red line.

**Figure 7:**
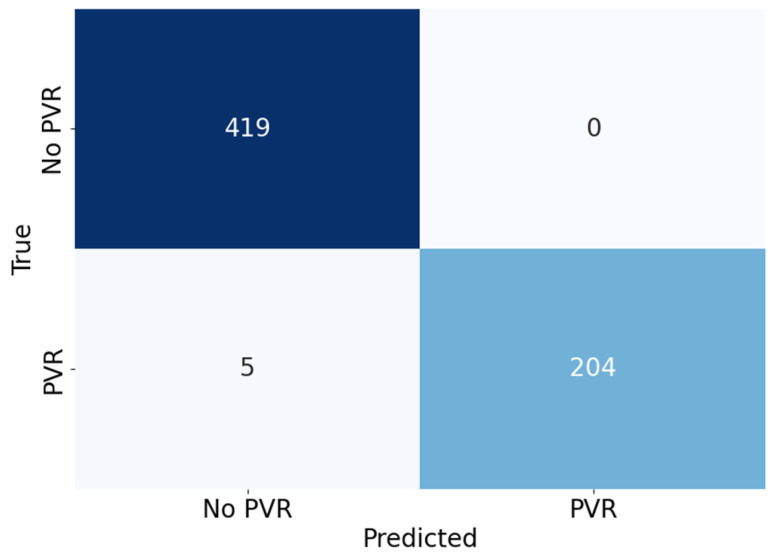
The confusion matrix shows the classification results, with only 5 out of 628 samples being incorrectly predicted, resulting in a 99.2% accuracy in predicting the correct classes.

Table 1 shows the comparison of the proposed segmentation path with conventional CNN-based segmentation networks. U-Net, which is commonly used as a baseline for medical image segmentation, achieved an average Dice coefficient of 0.913 with a standard deviation of 0.124. The implemented U-Net had 8.56 million parameters and a throughput of 1.33 frames per second (FPS) in the SoC environment. In comparison, the Attention U-Net achieved a much higher Dice coefficient of 0.944 on average with a standard deviation of 0.075 but had a slower throughput than the U-Net, despite having fewer parameters. The recently introduced BiSe-Netv2 showed even higher Dice coefficients than both U-Net and Attention U-Net (i.e., an average of 0.958 and a standard deviation of 0.034) with even fewer parameters. However, BiSeNetv2 was slower than U-Net, running at less than 1 FPS. In contrast, the proposed method had significantly fewer parameters (i.e., 0.97 million) and could be executed at a much faster rate of approximately 8 FPS, which was 5.96x , 52.87x, and 13.44x faster than the U-Net, Attention U-Net, and BiSeNetv2, respectively. Although the proposed method had a slightly lower Dice coefficient than BiSeNetv2 (i.e., 0.954 ± 0.045 vs. 0.958 ± 0.034, respectively), the segmentation results from the proposed network are promising, as shown in Figure 6. The validation accuracy of the classification path was over 0.99, indicating high accuracy in the classification results, as shown in the confusion matrix in Figure 7.

### 3.2. Evaluation of the Bladder Volume Measurement

**Figure 8:**
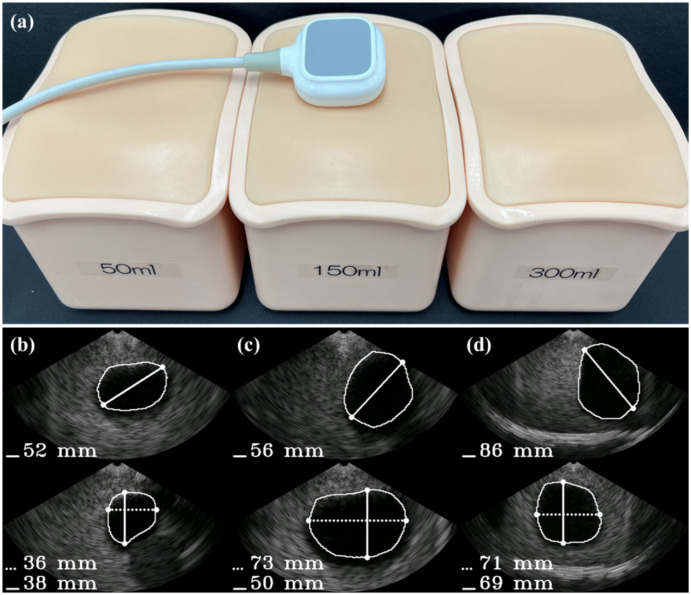
(**a**) Overall experimental setup for bladder volume measurement. Bladder phantoms with volumes of 50 mL, 150 mL, and 300 mL were measured using the integrated system, which combines the proposed method with a T-shaped array portable ultrasound system. (**b**–**d**) Measurement results for the 50 mL, 150 mL, 300 mL phantoms, respectively.

The bladder volume measurement results using the integrated system are depicted in Figure 8. To evaluate the system, the volume of an intravesical urine volume measurement phantom (US-16, Kyoto Kagaku, Kyoto, Japan) was measured using 30 separately acquired sagittal and transverse images for 50 mL, 150 mL, and 300 mL targets. The segmentation and classification paths were evaluated using the Dice coefficient and accuracy metrics, respectively. To quantitatively evaluate bladder volume measurements, automatic measurements were conducted five times on each phantom. The results in Table 2 show that the volumes could be calculated with less than 10% error when appropriate shape coefficients were selected. The coefficient of 0.72 was found to provide the most accurate measurement for both the 50 mL and 150 mL phantoms, while for the 300 mL phantom, the coefficient of 0.66 was most accurate. However, it is worth noting that when using the coefficient of 0.72 for the 300 mL phantom, a 5.78% error was observed, which is comparable to the 3.45% error observed when using the coefficient of 0.66. Therefore, using the coefficient of 0.72 resulted in measurements within tolerable error for all cases.

## 4. Discussion

An ultrasound-based bladder volume measurement is an effective method for detecting and managing PVR. To enhance its efficacy, an automatic bladder volume measurement method based on image analysis is necessary. Recent studies have demonstrated that deep learning-based image analysis techniques can be employed on ultrasound images for the management of urinary diseases [15,28]. However, conventional methods have high computational complexity, making them unsuitable for adoption on portable devices. As a result, it is challenging to integrate deep learning-based bladder volume measuring algorithms into portable systems.

In this study, a lightweight deep learning network was developed as a multitask network that performed classification and segmentation simultaneously. The multitask network has several advantages compared to networks that perform only segmentation or classification. In terms of computational complexity, the integrated device can save power or resources by using the classification node. For example, when the classification results show that images do not have ROIs, the post-processing algorithm for measurement does not need to be executed. In terms of accuracy and user experience, the classification node is also helpful. For instance, if the classification result indicates that an image does not have an ROI, the segmentation result is invalid and inaccurate and should not be presented to the users. These advantages make the multitask network more suitable for real clinical situations.

The segmentation accuracy of this network was validated by comparing it with conventional segmentation networks (U-Net, Attention U-Net, BiSeNetv2) using the Dice coefficient as the evaluation metric. Compared to the baseline U-Net, the Attention U-Net exhibited a significantly higher Dice coefficient of 0.944 with fewer parameters (7.91 M vs. 8.56 M). Although the recently introduced BiSeNetv2 had an even higher Dice coefficient with a smaller number of parameters, it still exhibited slow execution times in low-resource SoC environments (0.59 FPS). The slow execution time of these conventional networks could be problematic, particularly since two images (sagittal and transverse) were used to measure bladder volume automatically.

Compared to conventional networks, the developed network had fewer parameters (<1 M), and its Dice coefficient (0.954) was comparable to that of BiSeNetv2 (0.958) and higher than that of Attention U-Net and U-Net at 0.944 and 0.913, respectively. The drop in accuracy can be considered negligible, as the annotation process is performed by humans and may have labeling noise. Moreover, the accuracy of the proposed network is sufficient to be used for automatic volume calculation, as demonstrated in the previous section.

To efficiently implement the developed network on SoCs, further optimization based on quantization was carried out. After quantization, the proposed network could execute on SoCs at 7.93 FPS, enabling it to measure bladder volume approximately four times per second. Moreover, the classification accuracy of the proposed network was over 0.99, making it an efficient network with high accuracy for both PVR detection and bladder segmentation. The proposed network was also used in an end-to-end automatic algorithm to measure bladder volume. The algorithm accurately identified the axes (i.e., height, width, and depth) of the bladder, and with the proper shape coefficient, the bladder volume could be estimated within ±6% error of the actual volume. In addition, since the model was trained on dataset with various size and location of ROIs, the model can estimate ROIs regardless of their size or location.

While this study demonstrated the potential of the deep learning-based automatic bladder volume measurements on an SoC, there are still several limitations that need to be addressed. First, this study was focused on phantom studies and was not validated in in vivo cases. In further works, data from in vivo cases will be collected, and the clinical impact of the proposed method will be evaluated. Additionally, this study estimated the bladder volume using shape coefficients, but as shown in Table 2, improper shape coefficients can result in significant errors in volume estimations. To address this issue, future studies may investigate automatic estimation of bladder volume or shape coefficient. For example, a study may be conducted on deep learning methods that utilize prior knowledge of bladder shape to perform end-to-end volume prediction.

## 5. Conclusions

In this study, a lightweight deep learning network was developed to measure bladder volume on portable bladder ultrasound devices. The designed network showed comparable accuracy to conventional deep learning methods in terms of the Dice coefficient. Additionally, the execution of the designed network was much faster than that of conventional methods. An automatic axis detection algorithm was also utilized, and the bladder volume could be end-to-end automatically measured with under ±6% error with proper shape coefficients. Finally, the proposed network and algorithm were successfully integrated into a low-resource SoC-based portable bladder ultrasound system. However, further validation on in vivo cases and automatic estimation of shape coefficients may be necessary for future studies.

## Figures and Tables

**Table 1:** Comparison of the Segmentation Results.

	Dice Coefficient	# of Parameters	Throughput (FPS)
U-Net	0.913 ± 0.124	8.56 M	1.33
Attention U-Net	0.944 ± 0.075	7.91 M	0.15
BiSeNetv2	0.958 ± 0.034	3.12 M	0.59
Ours	0.954 ± 0.045	0.97 M	7.93

**Table 2:** Quantitative Evaluation Results of the Volume Measurement using Integrated System.

	Coefficient	50 mL	150 mL	300 mL
Unknown	0.72	50.89	157.45	317.34
Triangular prism	0.66	46.57	141.86	289.66
Cylinder	0.81	56.85	171.06	350.57
Cuboid	0.89	61.69	184.34	402.86
Spherical	0.52	37.84	112.29	233.06

## Data Availability

The data that support the findings of this study are available from the corresponding author upon reasonable request.
